# Quantitative Variation in m.3243A > G Mutation Produce Discrete Changes in Energy Metabolism

**DOI:** 10.1038/s41598-019-42262-2

**Published:** 2019-04-08

**Authors:** Ryan P. McMillan, Sidney Stewart, James A. Budnick, Clayton C. Caswell, Matthew W. Hulver, Konark Mukherjee, Sarika Srivastava

**Affiliations:** 1 Department of Human Nutrition, Foods and Exercise, Virginia Tech, Blacksburg, VA 24061 USA; 2 Metabolic Phenotyping Core at Virginia Tech, Blacksburg, VA 24061 USA; 3 Fralin Biomedical Research Institute at Virginia Tech Carilion, Roanoke, VA 24016 USA; 4 Department of Biomedical Sciences and Pathobiology, Center for One Health Research, VA-MD College of Veterinary Medicine, Virginia Tech, Blacksburg, VA 24060 USA; 5 Fralin Biomedical Research Institute at Virginia Tech Carilion, Roanoke, VA 24016 USA; 6 Present Address: Edward Via College of Osteopathic Medicine, Auburn, AL 36832 USA

## Abstract

Mitochondrial DNA (mtDNA) 3243A > G *tRNALeu*^*(UUR)*^ heteroplasmic mutation (m.3243A > G) exhibits clinically heterogeneous phenotypes. While the high mtDNA heteroplasmy exceeding a critical threshold causes mitochondrial encephalomyopathy, lactic acidosis with stroke-like episodes (MELAS) syndrome, the low mtDNA heteroplasmy causes maternally inherited diabetes with or without deafness (MIDD) syndrome. How quantitative differences in mtDNA heteroplasmy produces distinct pathological states has remained elusive. Here we show that despite striking similarities in the energy metabolic gene expression signature, the mitochondrial bioenergetics, biogenesis and fuel catabolic functions are distinct in cells harboring low or high levels of the m.3243 A > G mutation compared to wild type cells. We further demonstrate that the low heteroplasmic mutant cells exhibit a coordinate induction of transcriptional regulators of the mitochondrial biogenesis, glucose and fatty acid metabolism pathways that lack in near homoplasmic mutant cells compared to wild type cells. Altogether, these results shed new biological insights on the potential mechanisms by which low mtDNA heteroplasmy may progressively cause diabetes mellitus.

## Introduction

Mitochondria are the energy powerhouse of eukaryotic cells as they metabolize nutrients to produce adenosine triphosphate (ATP)^1^. Genetic mutations in the mitochondrial DNA (mtDNA) or nuclear DNA (nDNA) encoding oxidative phosphorylation (OXPHOS) genes that impair ATP production cause mitochondrial disorders^2^. Mitochondrial 3243A > G *tRNALeu*^*(UUR)*^ (m.3243A > G) heteroplasmic (i.e. wild type and mutant mtDNAs coexist) mutation is reportedly the most common pathogenic mtDNA mutation^3^. This mutation exhibits clinical heterogeneity, i.e. the high mtDNA heteroplasmy exceeding a critical threshold (typically > 85%) causes the MELAS syndrome^4^ whereas the low mtDNA heteroplasmy (typically < 45%) causes the MIDD syndrome^5,6^. Although the MELAS clinical onset occurs most often during childhood, the MIDD onset occurs at ~35–40 years of age and exhibits impaired insulin secretion with or without sensorineural deafness^7,8^.

In diabetic subjects, the mtDNA heteroplasmy levels have been reported to be in the range of ~1–45% in leukocytes and ~10–45% in pancreatic β-cells^9–11^. Surprisingly, subjects harboring ~0.01–0.1% mtDNA heteroplasmy levels also develop diabetes mellitus (DM) indicating a high penetrance of this mutation^12,13^. MtDNA heteroplasmy inversely correlates with the age of onset of MIDD, and DM can be either type I or type II in nature^7,14^. Interestingly, the paternal co-inheritance of type II DM in combination with a high mtDNA heteroplasmy in muscle was reported in a subject with MIDD who clinically evolved into MELAS syndrome over time^15^. A marked reduction in glucose stimulated insulin secretion (GSIS) has been reported in m.3243A > G carriers compared to the non-carriers^7^. Notably, mechanisms other than the autoimmune destruction have been implicated in β-cell loss and total islet mass reduction in the m.3243A > G mutation subjects exhibiting an impaired insulin secretion^16^.

To better understand the mechanisms of DM pathogenesis, we examined the mitochondrial metabolic gene expression profiles and function in human osteosarcoma-derived transmitochondrial cybrid cells carrying either a low or high level of m.3243A > G mutation compared to the wild type mtDNA containing osteosarcoma cells (143BTK^−^). We found that although the gene expression profiles of mitochondrial OXPHOS, TCA (tri-carboxylic acid) cycle, and FAO (fatty acid oxidation) pathways exhibits marked similarities, the mitochondrial energy metabolic function is discrete in cells harboring either a low or high level of the m.3243A > G mutation compared to wild type cells. Specifically, we demonstrate that low heteroplasmic mutant cells exhibit a significantly increased mitochondrial bioenergetics, complex IV activity, mtDNA content, endogenous levels of mtDNA encoded transcripts and OXPHOS proteins, as well as an enhanced rate of glucose, pyruvate, and fatty acid oxidation in contrast to the near homoplasmic mutant cells that show a significant mitochondrial functional impairment compared to wild type cells. We further demonstrate that a coordinate induction of transcriptional regulators of the mitochondrial biogenesis, glucose and fatty acid metabolism pathways underlie the enhanced energy metabolic phenotype of low heteroplasmic mutant cells in contrast to the near homoplasmic mutant cells that lack such a response compared to wild type cells. Our data suggests that the nuclear genome differentially perceives low and high levels of the m.3243A > G mutation and mount a discrete energy metabolic response shedding new insights on the potential mechanisms that may underlie DM pathogenesis.

## Results

### Low and high mtDNA mutation load generates a markedly similar gene expression signature for energy metabolic pathways

The generation of human transmitochondrial cybrid cells carrying ~30% or ~100% of the m.3243A > G mutation load, and their complete mtDNA sequence has been previously described^17^. Previous studies reported that the parental 143BTK^−^ osteosarcoma cells serve as a normal representative control for osteosarcoma derived cybrid cells carrying a variety of mtDNA heteroplasmic or homoplasmic mutations^18–24^. The potential effects of different mtDNA haplogroups on respiratory chain function were previously compared between the 143BTK^−^ cells and cybrid cells carrying a variety of mtDNA haplogroups from human control fibroblasts, and no significant differences were observed^25^. Furthermore, it has been shown that mitochondrial bioenergetics, ATP production, OXPHOS enzyme complex activities and assembly in 143BTK^−^ cells are not significantly different from the osteosarcoma derived cybrid cells harboring mtDNA from subjects with no history of mitochondrial diseases^21^. Therefore, the parental 143BTK^−^ cells were utilized as a normal reference control for this study.

**Figure 1:**
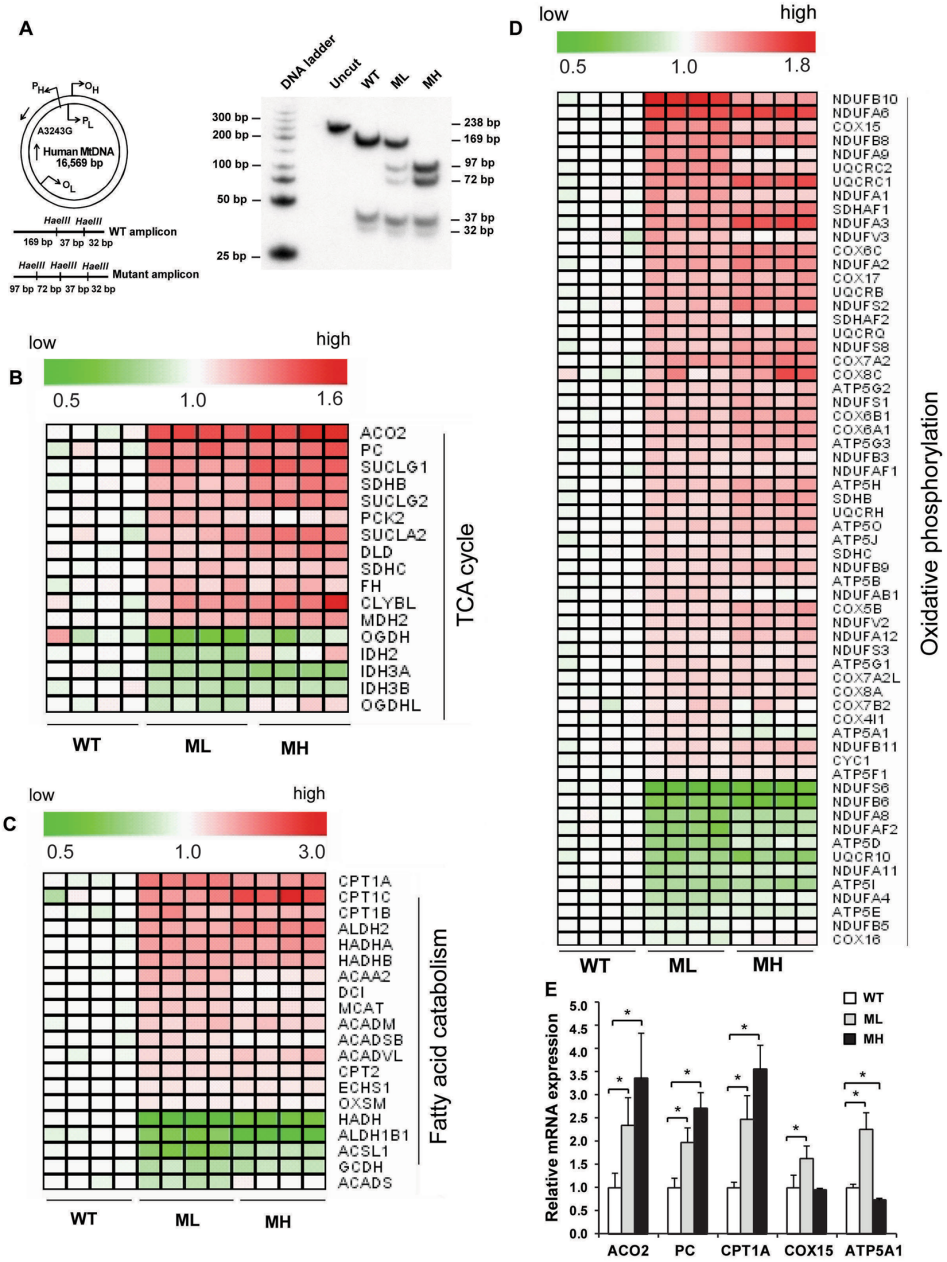
m.3243A > G *tRNA*^*Leu(UUR)*^ mutation alters the gene expression signature of energy metabolic pathways. (**A**) ‘Last cycle hot’ PCR/RFLP analysis showing the m.3243A > G heteroplasmy levels in WT, ML and MH cells. (**B–D**) Representative heat maps showing the gene expression profiles of TCA cycle, FAO and OXPHOS pathways in WT, ML and MH cells. Genes that are upregulated or downregulated are color coded in red or green, respectively. p < 0.05. (**E**) Relative mRNA expression of target genes in WT, ML and MH cells under HG conditions. Error bars are ± SD (n = 4). *p < 0.05.

To verify mtDNA heteroplasmy levels in the transmitochondrial cybrids, we performed a ‘last cycle hot’ PCR/RFLP analysis as described previously^26,27^. Quantitation of the band intensities from this analysis (after a short exposure of radioactive gel to X-ray film) revealed that low heteroplasmic mutant cells (henceforth referred as ‘ML’) harbored ~33% mutant and ~67% wild type mtDNA content whereas near homoplasmic mutant cells (henceforth referred as ‘MH’) harbored ~100% mutant mtDNA content compared to the parental 143BTK^−^ cells that harbored 100% wild type mtDNA (henceforth referred as ‘WT’; Fig. 1A). It is notable that we did not detect any wild type mtDNA in MH cells compared to WT cells even after an overnight exposure of the gel to X-ray film (Supplementary Fig. S1). To determine how a variable m.3243A > G mutation load impacts energy metabolic pathways, we compared the gene expression profiles of WT, ML and MH cells using the Affymetrix GeneChip HG U133 Plus 2.0 array (Affymetrix, Santa Clara, CA). Microarray analysis revealed that out of a total of ~20,000 genes, ~19% genes were upregulated and ~5% genes were downregulated in ML cells (p < 0.05) whereas ~35% genes were upregulated and ~6.6% genes were downregulated in MH cells (p < 0.05) compared to WT cells (data not shown). To examine the differential changes in energy metabolic pathway genes, we performed the KEGG (Kyoto Encyclopedia of Genes and Genomes) pathway analysis. This analysis revealed that the mitochondrial OXPHOS, TCA cycle, and FAO pathway genes were differentially regulated in ML and MH cells compared to WT cells (Fig. 1B–D), and a majority of the genes were significantly upregulated in both mutant cell types (p < 0.05). Interestingly, the gene expression signature appeared markedly similar between ML and MH cells compared to WT cells (Fig. 1B–D). Using a quantitative PCR (qPCR) analysis, we validated the gene expression changes for select target genes and found that the transcript levels of aconitase 2 (*ACO2*), pyruvate carboxylase (*PC*) and carnitine palmitoyl transferase 1 A (*CPT1A*) were significantly upregulated in ML and MH cells compared to WT cells. Additionally, the cytochrome *c* oxidase subunit 15 (*COX15*) and ATP synthase alpha subunit 1 (*ATP5A1*) transcripts were significantly upregulated in ML cells, whereas *ATP5A1* transcript was significantly downregulated in MH cells compared to WT cells (Fig. 1E). Altogether, our data suggests that both low and high levels of the m.3243A > G mutation alter expression of the energy metabolic pathway genes.

### Low and high mtDNA mutation load triggers a distinct mitochondrial bioenergetics response

**Figure 2:**
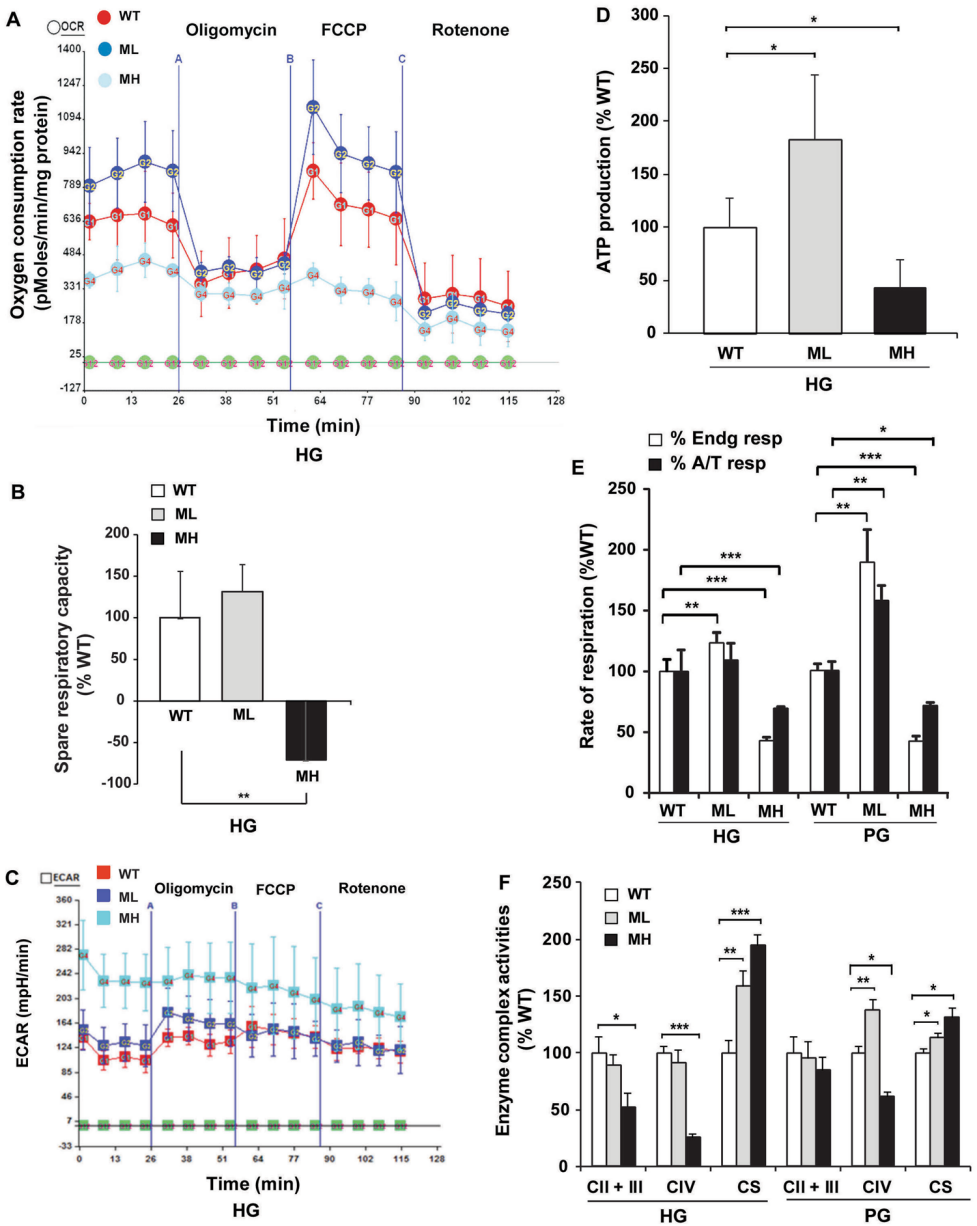
Quantitative differences in m.3243A > G mutation load produces a distinct bioenergetics phenotype. (**A**) Oxygen consumption measurements in WT, ML and MH cells under HG conditions using the Seahorse assay (n = 5). (**B**) Spare respiratory capacity measurements in WT, ML and MH cells under HG conditions (n = 5). (**C**) Extracellular acidification rate (ECAR) measurements in WT, ML and MH cells under HG conditions (n = 5). (**D**) ATP production measurements in WT, ML and MH cells under HG conditions (n = 5). Note: n = 5 represents five technical repeats for the representative Seahorse assay. The experiment was biologically repeated three times and similar results were obtained. (**E**) Endogenous and ascorbate/TMPD (A/T) driven respiration measurements in WT, ML and MH cells under HG and PG conditions using the Clark oxygen electrode (n = 3). (**F**) Spectrophotometric measurements of the OXPHOS enzyme complex II + III, complex IV and citrate synthase activities in WT, ML and MH cells under HG and PG conditions (n = 3). Data represented as %WT. Error bars are ± SD. *p < 0.05, **p ≤ 0.005, ***p ≤ 0.0005.

Next, we sought to determine the bioenergetics profile of WT, ML and MH cells using the Seahorse XF24 extracellular flux analyzer. Mitochondrial respiration was measured in the presence or absence of oligomycin (an ATP synthase inhibitor) and carbonyl cyanide 4-(trifluoromethoxy) phenylhydrazone (FCCP - a protonophore) followed by inhibition with rotenone (a complex I inhibitor). Interestingly, we observed that under high glucose (HG) culture conditions (i.e. ~25 mM glucose) the endogenous (or basal) and FCCP uncoupled respiration rates were significantly higher in ML cells compared to WT cells (Fig. 2A). As expected, MH cells showed a significant reduction in the endogenous respiration, FCCP uncoupled respiration, and spare respiratory capacity (i.e. a difference between the FCCP uncoupled and endogenous respiration) compared to WT cells (Fig. 2A,B). The ECAR (extracellular acidification rate) profile was not significantly altered in ML cells, however it was significantly higher (~2.1-fold) in MH cells compared to WT cells indicating their enhanced glycolytic rate (Fig. 2C and Supplementary Fig. S2). To determine whether the enhanced respiration in ML cells was coupled to ATP production, we measured the ratio of endogenous to oligomycin-inhibited respiration (E/OL), the ratio of FCCP uncoupled to endogenous respiration (FCCP/E), and the difference between endogenous and oligomycin-inhibited respiration (a measure of ATP production) compared to WT cells. We observed no significant changes in E/OL and FCCP/E ratios (Supplementary Fig. S3), and a significantly higher ATP production in ML cells compared to WT cells (Fig. 2D) suggesting that enhanced respiration is coupled to ATP production in ML cells. In contrast, MH cells showed a significant decrease in FCCP/E ratio and ATP production compared to WT cells suggesting that respiration is uncoupled from ATP production in these cells (Fig. 2D and Supplementary Fig. S3). In addition to the Seahorse assay, total cellular respiration was measured using a Clark oxygen electrode. This assay further verified that ML cells exhibit a significant increase in endogenous respiration (i.e. measured in the absence of exogenously added substrates) whereas MH cells display a significant decrease in endogenous respiration and ascorbate/TMPD (A/T) or complex IV substrate driven respiration compared to WT cells under HG culture conditions (Fig. 2E). Next, we examined the cellular oxidative capacity by measuring respiration under low glucose (i.e. physiological glucose (PG), ~5.5 mM) culture conditions. Because both the Seahorse and the Clark oxygen electrode assays showed similar results for endogenous respiration measurements under HG conditions, the Clark electrode assay was utilized for respiration measurements under PG conditions. Interestingly, we found that ML cells exhibited ~2-fold increase in endogenous respiration and ~50% increase in A/T driven respiration compared to WT cells under PG conditions indicating an increase in their oxidative capacity and complex IV respiration, respectively (Fig. 2E). In contrast, MH cells exhibited ~58% decrease in endogenous respiration and ~29% decrease in A/T driven respiration compared to WT cells under PG conditions indicating their reduced oxidative capacity and complex IV respiration, respectively (Fig. 2E). This result was further corroborated by our findings that complex IV enzyme activity is enhanced by ~50% in ML cells and reduced by ~40% in MH cells compared to WT cells under PG conditions (Fig. 2F). Citrate synthase activity was significantly higher in ML and MH cells compared to WT cells under both HG and PG conditions indicating enhanced TCA cycle activity in both the mutant cell types (Fig. 2F). Altogether, our results suggest that low and high levels of the m.3243A > G mutation generates distinct responses to mitochondrial bioenergetics and oxidative capacity.

### Low mtDNA mutation load strongly induces mitochondrial biogenesis

**Figure 3:**
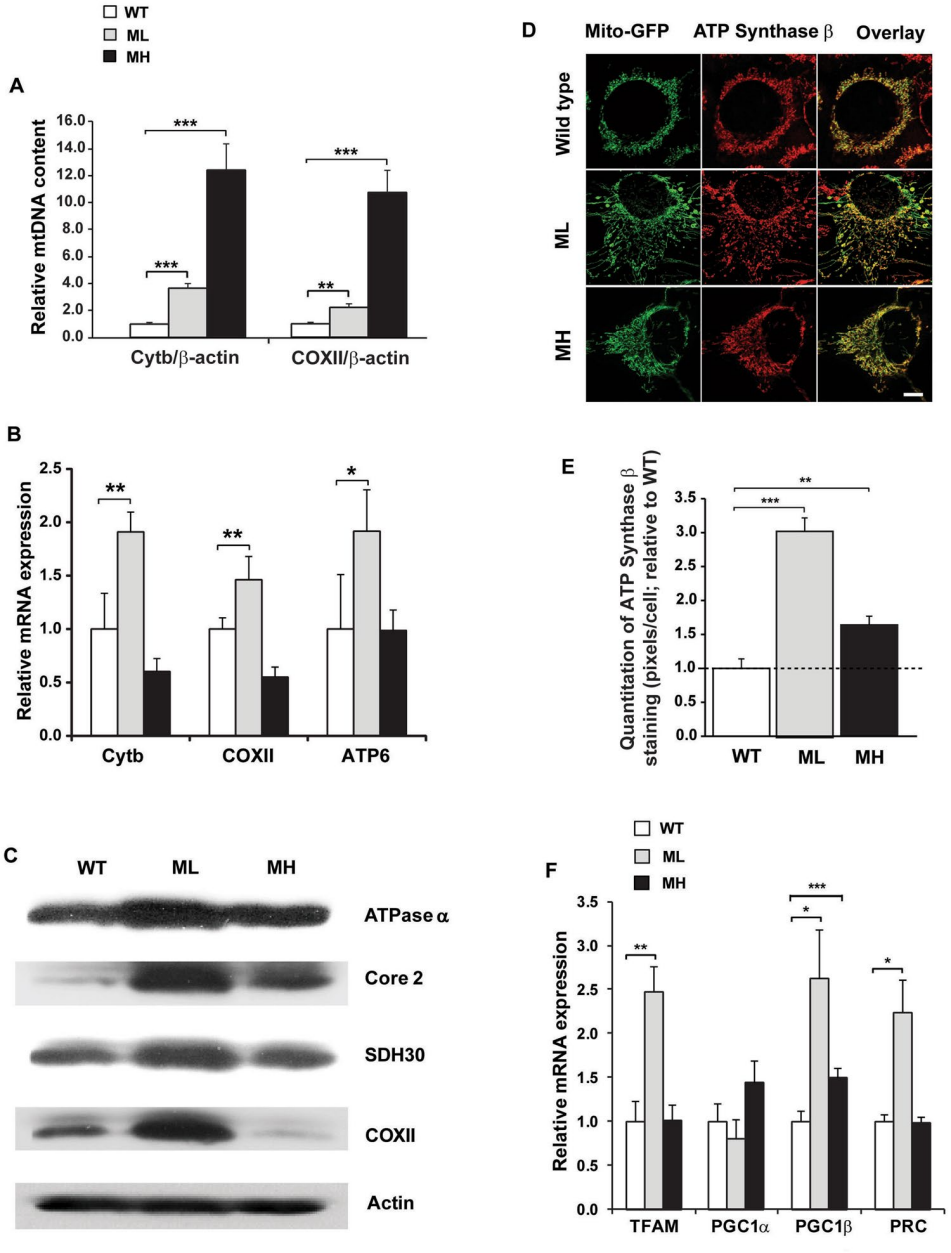
Low heteroplasmic m.3243A > G mutation strongly induces mitochondrial biogenesis. (**A**) Relative mtDNA to nuclear DNA ratio in WT, ML and MH cells under HG conditions (**B**) Relative expression levels of mtDNA encoded transcripts in WT, ML and MH cells under HG conditions. (**C**) Representative Western blot showing the steady-state levels of OXPHOS subunits in WT, ML and MH cells under HG conditions. The full-length blot is shown in Supplementary Fig. S4 (**D**) Lentiviral mediated mito-GFP transduction and ATP synthase β immunostaining in WT, ML and MH cells under HG conditions. (**E**) Quantitation of ATP synthase β staining from ~200 cells across six biological replicates in WT, ML and MH cells. (**F**) Relative mRNA expression levels of TFAM, PGC-1α, PGC-1β and PRC in WT, ML and MH cells under HG conditions. Error bars are ± SD (n = 4). *p < 0.05, **p ≤ 0.005, ***p ≤ 0.0005.

We next sought to determine whether the increased OXPHOS in ML cells was associated with enhanced mitochondrial biogenesis. To examine mitochondrial biogenesis, we first measured the steady-state levels of mtDNA and mtRNA by qPCR. Interestingly, we found that the ratio of mtDNA to nuclear DNA was significantly higher in both the ML cells (~2.2–3.7-fold) and MH cells (~10.7–12.4-fold) compared to WT cells suggesting an increased mtDNA proliferation in both mutant cell types (Fig. 3A). The steady-state level of mtDNA encoded transcripts i.e. cytochrome *b* (*Cytb)*, cytochrome *c* oxidase subunit II (*COXII*) and ATP synthase subunit 6 (*ATP6*) were significantly upregulated (~1.5–2.0-fold) in ML cells whereas no significant change in these transcript levels were found in MH cells compared to WT cells (Fig. 3B). Next, we examined the steady-state levels of OXPHOS proteins by Western blot analysis and found that multiple OXPHOS proteins including the COXII and ATPase α catalytic subunits of complex IV and V respectively were upregulated in ML cells, whereas COXII subunit was reduced in MH cells compared to WT cells (Fig. 3C). Next, we examined the mitochondrial mass by transducing the cells with lentiviral particles containing a mitochondrially-targeted green fluorescent protein (mito-GFP) and also by immunostaining the cells using an ATP synthase β subunit antibody. Confocal microscopy analysis revealed that ML cells harbor a potent induction in mitochondrial mass whereas MH cells display a moderate induction in mitochondrial mass compared to WT cells (Fig. 3D). Moreover, the mitochondrial staining pattern appeared punctate in WT cells, whereas changes in mitochondrial morphology were observed in both ML and MH cells with many mitochondria exhibiting elongated structures and reticular network suggesting that mitochondrial dynamics may be altered in the mutant cells (Fig. 3D). Quantitation of the ATP synthase β staining from ~200 cells for each sample revealed a ~3-fold increase in mitochondrial mass in ML cells and ~1.5-fold increase in mitochondrial mass in MH cells compared to WT cells (Fig. 3E). Finally, we verified the steady-state levels of transcriptional regulators of the mitochondrial biogenesis pathway by qPCR analysis since multiple probe sets for the genes on the Affymetrix GeneChip microarray exhibited discordant results^28^ (data not shown). Importantly, we found that the transcript levels of mitochondrial transcription factor A (TFAM) and two members of the peroxisome proliferator-activated receptor γ coactivator 1 (PGC-1) family (i.e. PGC-1β and PGC-1 related co-activator or PRC) were significantly elevated (~2.0–2.5-fold) in ML cells compared to WT cells (Fig. 3F). In contrast, only the PGC-1β transcript was significantly upregulated (~40%) in MH cells compared to WT cells (Fig. 3F). These results suggest that the low m.3243A > G mutation load triggers a coordinate induction of factors that regulate expression of the mtDNA genome and the nDNA encoded mitochondrial genes which could then act cooperatively to stimulate mitochondrial biogenesis. In contrast, the high m.3243A > G mutation load causes a potent mtDNA proliferation and an increased PGC-1β expression which could stimulate mitochondrial mass (as a compensatory response), but fails to induce the mtDNA gene expression and mitochondrial function possibly due to a severe mitochondrial translational defect and a relatively high number of poor quality mitochondria, respectively. Moreover, the increased COXII catalytic subunit level in ML cells may stimulate the complex IV activity and respiration, whereas a reduced COXII subunit level in MH cells may impair complex IV activity and respiration, respectively.

### Low and high mtDNA mutation load produces discrete changes in fuel catabolism

**Figure 4:**
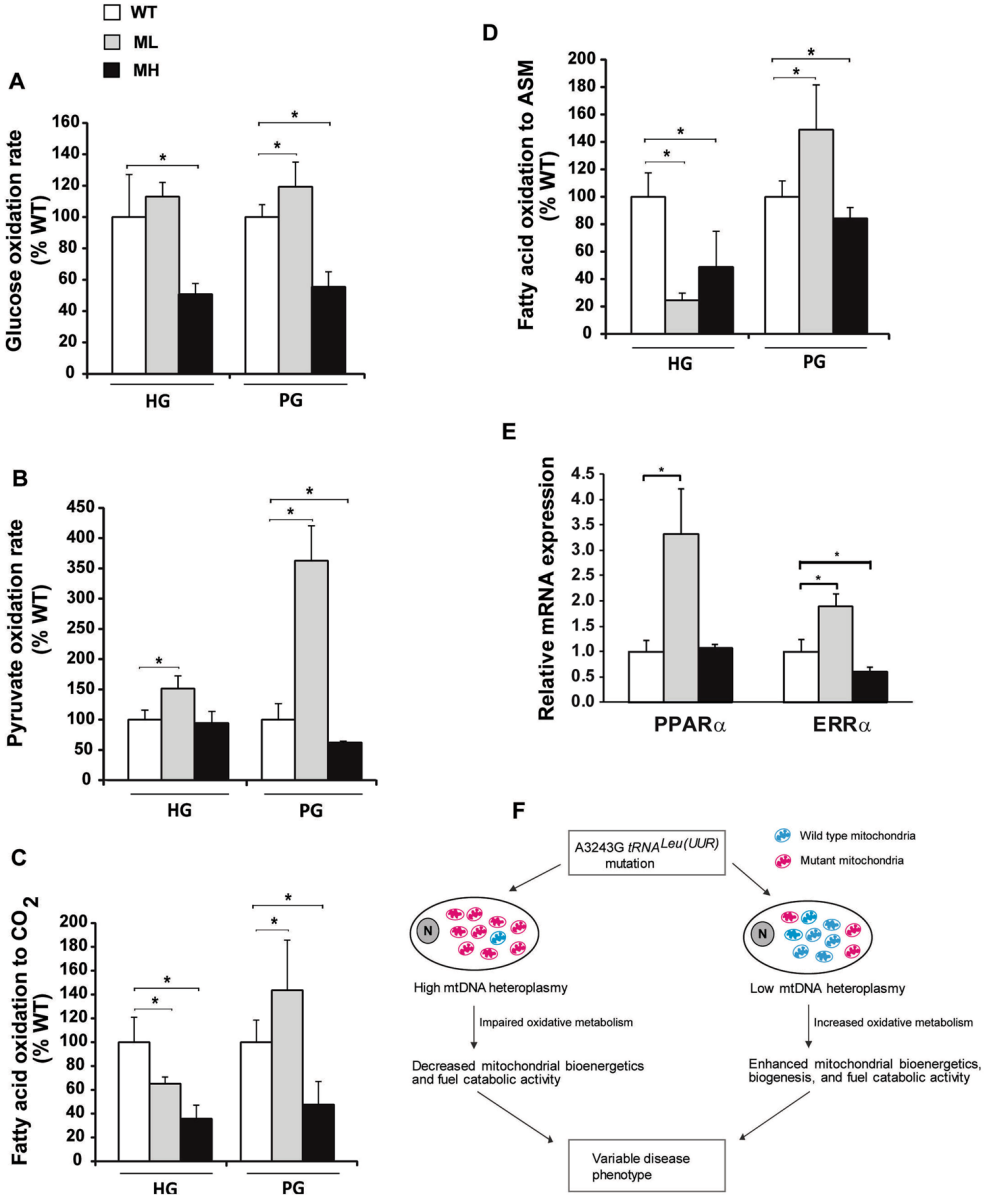
Quantitative differences in m.3243A > G mutation load generates discrete changes in fuel catabolism. (**A**) Measurements of glucose oxidation in WT, ML and MH cells under HG and PG conditions (n = 5). (**B**) Measurements of pyruvate oxidation in WT, ML and MH cells under HG and PG conditions (n = 5). (**C**) Complete FAO measurements in WT, ML and MH cells under HG and PG conditions (n = 5). (**D**) Incomplete FAO measurements in WT, ML and MH cells under HG and PG conditions (n = 5). (**E**) Relative mRNA expression levels of PPARα and ERRα in WT, ML and MH cells under HG conditions (n = 4). Data represented as %WT. Error bars are ± SD. *p < 0.05. (**F**) Schematic diagram depicting the enhanced energy metabolic phenotype in ML cells and reduced energy metabolic phenotype in MH cells that may differentially impact cellular processes in a tissue-specific manner leading to a variable disease expression. Wild type mitochondria are represented in cyan color, mutant mitochondria in magenta color, and ‘N’ represents the nucleus.

Finally, we sought to determine how a variable m.3243A > G mutation load impacts fuel catabolism. First, we examined the cellular glucose and pyruvate oxidation by measuring the production of ^14^CO_2_ from the oxidation of radiolabeled [U-^14^C]-glucose and [1-^14^C]-pyruvate respectively as described^29,30^. We found that under HG conditions the ML cells exhibit ~50% increase in pyruvate oxidation whereas under PG conditions they display a significant increase in both pyruvate oxidation (~3.5-fold) and glucose oxidation (~20%) compared to WT cells (Fig. 4A,B). In contrast, MH cells exhibit ~50% decrease in glucose oxidation compared to WT cells under both HG and PG conditions (Fig. 4A). Furthermore, MH cells display a significantly reduced pyruvate oxidation (~38%) compared to WT cells under PG conditions (Fig. 4B). Next, we examined the fatty acid oxidation (FAO) by measuring the production of ^14^CO_2_ (complete FAO) and ^14^C-labeled acid-soluble metabolites (incomplete FAO) from the oxidation of radiolabeled [1-^14^C]-palmitate as described^29,30^. We observed that under HG conditions, both the complete and incomplete FAO rates were significantly lowered in ML and MH cells compared to WT cells (Fig. 4C,D) indicating that the mutant cells preferentially utilize glucose over fatty acids as an energy source under HG conditions. Also, glucose provision is known to inhibit FAO – the ‘Randle cycle’ concept^31^. Interestingly, under PG conditions, the complete and incomplete FAO rates were significantly enhanced by ~43% and ~48% respectively in ML cells compared to WT cells (Fig. 4C,D). However, in MH cells the complete and incomplete FAO rates were significantly reduced by ~52% and ~16% respectively compared to WT cells under PG conditions (Fig. 4C,D). To further examine the underlying cause of abnormal fuel catabolism in ML and MH cells, we determined the steady-state levels of transcriptional regulators that control fatty acid and glucose metabolism pathways i.e. the peroxisome proliferator-activated receptor alpha (PPARα) and the estrogen-related receptor α (ERRα), respectively. Because multiple probe sets for the *PPAR*α and *ERR*α genes revealed discordant results on the Affymetrix GeneChip microarray (data not shown), we performed a qPCR analysis to verify their transcript levels. Interestingly, we observed a significant upregulation of the PPARα (~3-fold) and ERRα (~2-fold) transcripts in ML cells compared to WT cells whereas the ERRα transcript was significantly downregulated (~50%) in MH cells compared to WT cells (Fig. 4E). Altogether, our data suggests that low levels of the m.3243A > G mutation stimulates glucose, pyruvate and fatty acid catabolism whereas high levels of the m.3243A > G mutation impairs fuel catabolism under PG conditions, and that the induced PPARα and ERRα levels may be underlying factors for the abnormally high rates of fuel breakdown in ML cells.

## Discussion

MIDD and MELAS syndromes represent the two most frequent clinical outcomes of the m.3243A > G heteroplasmic mutation. Although a defective energy metabolism is implicated in the MELAS pathogenesis, the mechanisms underlying DM pathogenesis remains poorly understood. Previous studies examining the effects of m.3243A > G mutation in cybrids were performed under high glucose conditions^17,32^. Notably, the concentrations of glucose above 10 mM are analogous to a diabetic condition within the cell culture system. Because high glucose mainly drives the anaerobic glycolysis and suppresses the aerobic OXPHOS function, we investigated the energy metabolic function of cybrid cells under the physiological glucose conditions. Our findings demonstrate that ML and MH cells exhibit a similar transcriptional signature for the energy metabolic pathway genes but display discrete changes in the oxidative metabolism. It is possible that because the mitochondrial biogenesis is enhanced (~3-fold) and a majority of mtDNA content (~67%) is wild type in ML cells, these factors concomitantly participate in enhancing the oxidative metabolism in ML cells. On the contrary, although mitochondrial biogenesis is enhanced (~1.5-fold) in MH cells along with an intense mtDNA proliferation (a compensatory response), nearly all of the mtDNA content (~100%) is mutated that could severely impair the oxidative metabolism. Increased mtDNA content was also previously reported in several tissues from the MELAS patients indicating that mitochondrial proliferation is ubiquitous and is not confined to ragged-red fibers in the muscle^33^. Our findings suggest that discrete changes in energy metabolism in response to a low or high m.3243A > G mutation load could differentially impact cellular processes in a tissue-specific manner leading to a variable disease expression (Fig. 4F). Notably, both ML and MH cells harbor the same mtDNA haplotype^17^ but exhibit discrete changes in oxidative metabolism indicating that the distinct effects likely arise due to a difference in their mtDNA mutation load. It is unlikely that the observed differences are due to nuclear genetic abnormalities as both the mutant cells and WT cells harbor an isogenic nuclear background^17^. Although the genomic instability is a hallmark of cancer cells, the 143BTK^−^ cells were reported to be the most genetically stable among various osteosarcoma cell lines with no chromosomal abnormalities observed after 275 doublings^34^. Our findings support a previously developed mathematical model predicting that cells with a subthreshold level of the m.3243A > G mutation tend to upregulate energy production pathways^35^. Additionally, our findings are consistent with a study implicating that up to ~64% m.3243A > G heteroplasmy levels exhibits a trend towards an increase in complex IV activity^36^.

Can enhanced oxidative metabolism contribute to DM pathogenesis? Mitochondria are the prime sites for the generation and integration of metabolic signals that couple glucose recognition with insulin secretion in pancreatic β-cells^37,38^. Mitochondria also generate several metabolic coupling factors that are necessary for amplifying and sustaining the GSIS response^37,39^. MtDNA depletion in pancreatic β-cells has been shown to impair the GSIS response emphasizing a critical role of mitochondria in the insulin secretion^40,41^. Alterations in the biochemical pathways that link glucose uptake with the ATP generation could thus alter the GSIS response. Our findings are in agreement with a previous study showing that mitochondrial respiration and OXPHOS enzyme activities are significantly enhanced during the initial stages of type I- and type II- DM in a rat model^42^. Interestingly, a higher plasma insulin concentration and rate sensitivity have been reported in the m.3243A > G carriers who had not clinically developed DM^43^. Moreover, in the clinical subjects, the resting metabolic rate is enhanced at a very early stage of DM, and is implicated in the progressive development of metabolic abnormalities^44^. Notably, the β-cell insulin secretion and the skeletal muscle maximal oxidative capacity were found to inversely correlate with the m.3243A > G heteroplasmy levels^43,45^. Additionally, genetic mutations that inactivate the ATP-sensitive potassium channel (K_ATP_) cause glucose intolerance and DM^46^, and a transgenic expression of the dominant negative K_ATP_ channel in mice increases the β-cell calcium transients causing cell death and DM^47^. It is therefore tempting to speculate that increased cellular ATP levels may cause a prolonged inactivation of the K_ATP_ channel resulting in glucose intolerance and/or DM. Increased FAO has also been shown to impair insulin secretion^48^, and incomplete FAO is a major cause of the mitochondrial distress and muscle insulin resistance^49^. In conclusion, it is possible that the enhanced oxidative metabolism involving increased ATP generation and/or increased FAO are initial underlying factors that participate in the pathogenesis of m.3243A > G associated DM.

## Methods

### Cell culture procedures

The generation of human osteosarcoma 143B(TK^−^)ρ° derived transmitochondrial cybrid cell lines harboring ~30% and ~100% of the m.3243A > G mutation load have been described previously^17^. These cybrid cells and the parental wild type 143BTK^−^ cells were obtained from Picard *et al*.^17^. Cells were cultured in a Dulbecco’s Modified Eagle’s Medium containing either a high glucose (i.e. 4.5 g/L or 25 mM) or low glucose (i.e. 1 g/L or 5.5 mM), and were supplemented with 1 mM pyruvate, 50 μg/ml uridine, and 10% fetal bovine serum, and incubated at 37° C in a 5% CO_2_ incubator.

### ‘Last cycle hot’ PCR/RFLP analysis

MtDNA heteroplasmy levels were determined by the ‘last cycle hot’ PCR/RFLP method as previously described^26,27^. Briefly, a 238 bp fragment was PCR amplified from the total DNA isolated from 143B wild type and the m.3243A > G mutant cybrid cell lines, and one round of primer extension was performed using the radiolabeled α[^32^P]dCTP nucleotide on the amplified fragments. The radiolabeled wild type and m.3243A > G mutated amplicons were then subjected to a *HaeIII* digestion followed by electrophoresis on a 12% polyacrylamide gel. Subsequently, the gel was dried using a gel dryer and subjected to autoradiography. The wild type amplicons contained two *HaeIII* enzyme sites whereas the m.3243A > G mutation in the mutant amplicons allowed the creation of an additional *HaeIII* site thus providing a diagnostic RFLP. The *HaeIII* digestion of the wild type amplicons generated three fragments (i.e. 169 bp, 37 bp and 32 bp) whereas that of the mutant amplicons generated four fragments (i.e. 97 bp, 72 bp, 37 bp and 32 bp), respectively. Band intensities were quantitated using the image J software (NIH, Bethesda, MA).

### Mitochondrial respiration measurements

Mitochondrial respiration was measured using both the Clark oxygen electrode and the Seahorse assays. For the Clark electrode assay, a respiration buffer containing 25 mM Tris HCl, 10 mM K_2_HPO_4_, and 150 mM sucrose was used to measure respiration in a water-jacketed cell magnetically stirred at 37° C (Hansatech instruments, Norfolk, UK), as described^26^. Briefly, the cells were cultured in a HG or LG media, and the rate of endogenous as well as ascorbate/TMPD (N,N,N′,N′-tetramethyl-p-phenylenediamine) or complex IV substrate driven respiration were measured in intact cells resuspended in the respiration buffer, using a Clark oxygen electrode. The total protein content in all samples was measured using the Bradford method^26^. For Seahorse assays, ~50 × 10^3^ cells were seeded in a XF24 plate and the sensor cartridge was equilibrated overnight with a XF24 calibrant solution in a non-CO_2_ incubator at 37 °C. On the next day, three injection ports were loaded with a 1 μg/ml oligomycin, 1 μM FCCP, and 1 μM rotenone, respectively. The sensors were calibrated and the assay was run on a Seahorse XF24 extracellular flux analyzer (Seahorse Bioscience, North Billerica, MA), as described^50^. The total protein content in all samples was measured using the Pierce^TM^ BCA protein assay kit (Thermo Fisher Scientific, Inc., MA, USA).

### OXPHOS complex activity measurements

All OXPHOS enzyme complex activities were measured spectrophotometrically using the total cell homogenates, as described^26,51^. Briefly, the succinate-cytochrome *c* reductase (complex II + III) and cytochrome *c* oxidase (complex IV) activities were measured at 37 °C by following a reduction or oxidation of cytochrome *c*, respectively, at 550 nm. Citrate synthase activity was monitored at 30 °C by measuring a production of the free 5-thio-2-nitrobenzoate anions, at 412 nm.

### Western blot analysis

Total cellular extracts (~100 μg) were run on the SDS-PAGE and the proteins were transferred to nitrocellulose membranes (Bio-Rad, CA). The blots were blocked overnight in a 5% milk, and incubated with a primary antibody for 3 hours followed by incubation with a secondary anti-mouse or anti-rabbit IgG conjugated with a horseradish peroxidase (HRP) for 1 hour. The chemiluminescent signal was detected using an ECL Western blotting detection kit (GE Healthcare, NJ). A cocktail of monoclonal antibodies against the ATPase α, SDH30, Core 2, and COXII were obtained from Abcam (Cambridge, MA). A polyclonal anti-actin antibody was obtained from Sigma (St. Louis, MO).

### Immunostaining

Cells were plated on the poly-L-lysine coated coverslips and transduced with a mitochondrially-targeted GFP lentivirus. Seventy-two hours post-transduction, the cells were washed with phosphate buffer saline (PBS), and fixed using a 4% paraformaldehyde made in PBS, for 20 minutes at room temperature (RT). Subsequently, the cells were washed with PBS followed by a permeabilization and blocking step using the 0.1% Triton X-100 and 5% goat serum in PBS, for 1 hour at RT. Cells were then incubated with a primary anti-ATP synthase β monoclonal antibody (1:200 dilution; Mitosciences, OR) for 1 hour at RT, followed by incubation with a secondary Alexa-633 anti-mouse antibody (1:250 dilution) for 30 minutes at RT. Coverslips were washed with PBS and mounted on glass slides using a VECTASHIELD antifade mounting medium (Vector Laboratories Inc., CA). Imaging was done using a 63X oil immersion objective on the confocal laser scanning microscope (ZEISS Axio Examiner.Z1 LSM 710), and a ZEN 2011 software was used for the image acquisition. For image quantitation, single high-power (63X) field images were obtained from the central part of six cover slips/sample. All images were anonymized and blindly analyzed using the Fiji (Image J) software. Images were threshold using the default settings, and pixels of labeled mitochondria were measured from the entire high-power field. Total number of cells in each field were counted manually using a cell counter function in the Fiji software. The number of mitochondria/cell in each image was derived by dividing the total mitochondrial pixels by the total number of cells. Post-analysis, the images were decoded and grouped into appropriate samples for a statistical analysis.

### Measurements of glucose, pyruvate and fatty acid oxidation

The pyruvate and glucose oxidation rates were determined by measuring ^14^CO_2_ production from the oxidation of [1-^14^C]pyruvate and [U-^14^C]glucose (American Radiolabeled Chemicals, MO), respectively, as described^29^. Complete and incomplete FAO rates were determined by measuring the ^14^CO_2_ production and ^14^C-labeled acid-soluble metabolites production, respectively, from the oxidation of [1-^14^C]palmitate (American Radiolabeled Chemicals, MO), as described^30^. Cells were lysed in 0.05% sodium dodecyl sulfate for protein estimation, and the data was normalized to total protein content.

### Quantitative PCR assays

For mRNA quantitation, the total cell RNA was isolated using a RNeasy kit (Qiagen, CA) and treated with a RNase free DNase (Promega, WI), followed by the cDNA synthesis using an iscript cDNA synthesis kit (Bio-Rad, CA). For mtDNA quantitation, the isolated total DNA (~10 ng) was used for the assay. Real-time SYBR green-based qPCR assays were performed using the mtDNA specific or the nuclear genes specific primers, on a LightCycler 480 instrument II (Roche, IN). The amount of transcripts or mtDNA in the samples was calculated using the absolute or relative quantitation method. Human β-actin or β-2-microglobulin (B2M) genes were used as the internal reference controls for normalization. All measurements were done in quadruplicates (n = 4). A list of the primer sequences is provided in Supplementary Table 1.

### Microarray analysis

Total cell RNA was isolated using the RNeasy kit and treated with an RNase free DNase. Microarray analysis was performed using the Affymetrix human GeneChip HG U133 Plus 2.0 array (Affymetrix, Santa Clara, CA). Four independent RNA isolations and microarray experiments were performed for each sample (n = 4). Microarray data processing and analysis were performed using two independent software programs: R Bio conductor (http://www.bioconductor.org)^52^ and FlexArray from Genome Quebec (Montreal, Quebec, Canada; http://genomequebec.mcgill.ca/FlexArray)^53^. Raw data from the array scans were normalized using the robust multi-chip average (RMA) method. Statistical significance of data was analyzed by a two-tailed Student’s t-test (p < 0.05). Pathway analyses were performed using the Ingenuity Pathway Analysis software (http://www.ingenuity.com) and the KEGG pathway database (http://www.genome.jp/kegg/pathway.html). The clustering and visualization were carried out using the MultiExperiment Viewer (MeV) (http://www.tm4.org). The expression patterns of differentially regulated genes were graphically represented in a heat map. The microarray data has been deposited in NCBI’s Gene Expression Omnibus and is accessible through GEO Series accession number GSE129091.

### Statistics

Unpaired two-tailed Student’s t-test were performed for statistical analysis to compare two different groups i.e. WT cells compared with either ML cells or MH cells. Data are represented as mean ± standard deviation (SD). Significant differences are noted as *p < 0.05, **p ≤ 0.005, ***p ≤ 0.0005.

## Supplementary information

Supplementary information

## Data Availability

All of the data are available upon request.
