# Supplementary material for: Quantitative Variation in m.3243A > G Mutation Produce Discrete Changes in Energy Metabolism
Source: Sci Rep. 2019 Apr 8;9:5752. doi: 10.1038/s41598-019-42262-2 (PMC6453956; doi:10.1038/s41598-019-42262-2)
Supplement: Supplementary file 1 — Supplementary information [file 41598_2019_42262_MOESM1_ESM.pdf]

Supplementary Information

Quantitative Variation in m.3243A>G Mutation Produce Discrete Changes in Energy Metabolism

Ryan P. McMillan,[1,2] Sidney Stewart,[3,†] James A. Budnick,[4] Clayton C. Caswell,[4] Matthew W. Hulver,[1,2] Konark Mukherjee,[3] and Sarika Srivastava,[5*]

[1]Department of Human Nutrition, Foods and Exercise, Virginia Tech, Blacksburg, VA 24061 USA

[2]Metabolic Phenotyping Core at Virginia Tech, Blacksburg, VA 24061, USA

[3]Fralin Biomedical Research Institute at Virginia Tech Carilion, Roanoke, VA 24016, USA

[4]Department of Biomedical Sciences and Pathobiology, Center for One Health Research, VA-MD College of Veterinary Medicine, Virginia Tech, Blacksburg, VA 24060, USA

[5]Fralin Biomedical Research Institute at Virginia Tech Carilion, Roanoke, VA 24016, USA. Sarika_Srivastava@vtc.vt.edu

[†]Present address: Edward Via College of Osteopathic Medicine, Auburn, AL 36832, USA

[*]Corresponding author

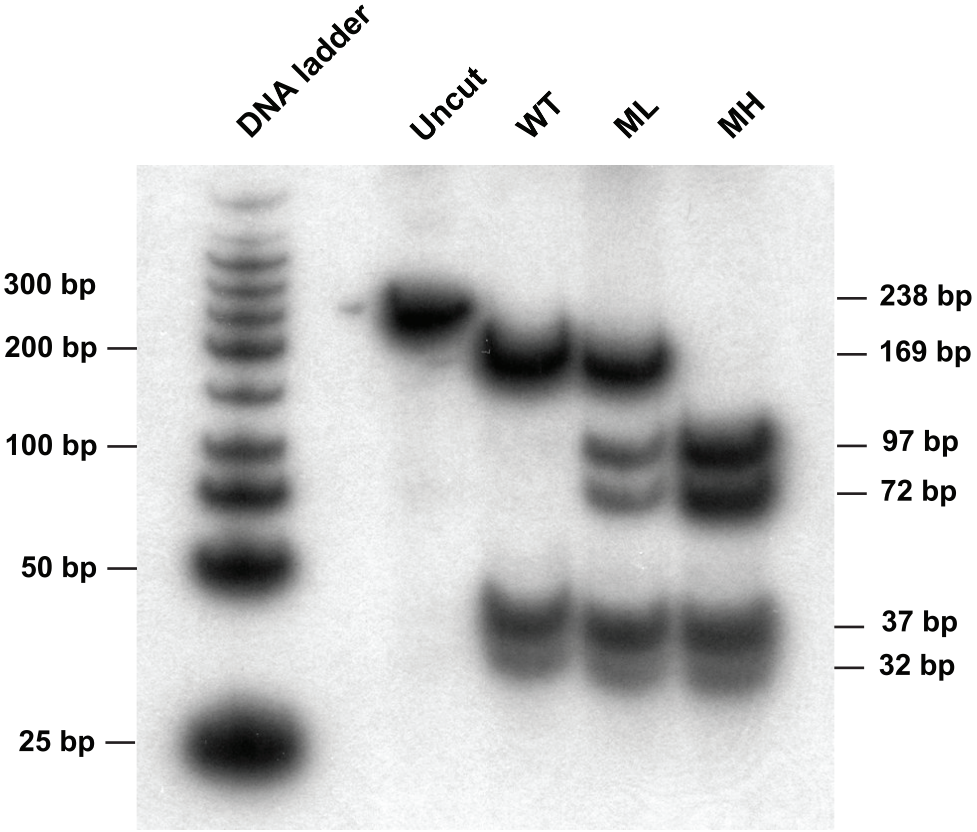

Figure S1. MtDNA heteroplasmy analysis. The 'last cycle hot' PCR/RFLP method was used to examine mtDNA heteroplasmy levels in WT, ML and MH cells. Note that even after an overnight exposure of the radioactive gel to X-ray film we did not detect a presence of wild type diagnostic fragment (i.e. 169 bp band) in MH cells compared to WT cells indicating that the m.3243A>G mutation is homoplasmic in MH cells.

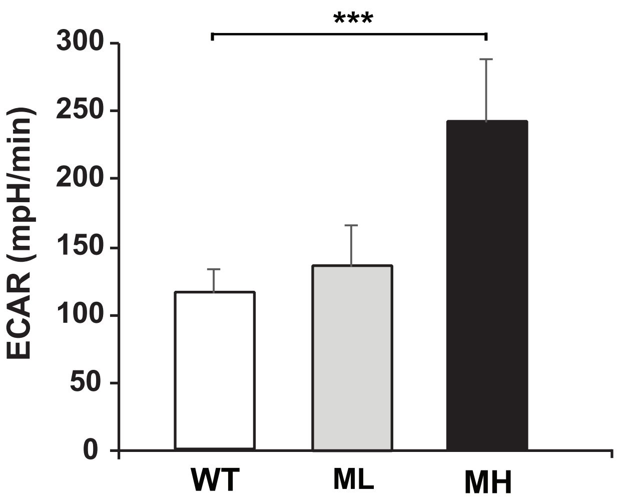

Figure S2. Quantitation of the extracellular acidification rate. The ECAR profile in WT, ML and MH cells obtained from the Seahorse analysis was quantitated. Note that MH cells exhibited ~2.1-fold increase in the ECAR compared to WT cells suggesting their enhanced rate of glycolysis. No significant change in the ECAR was observed in ML cells compared to WT cells.

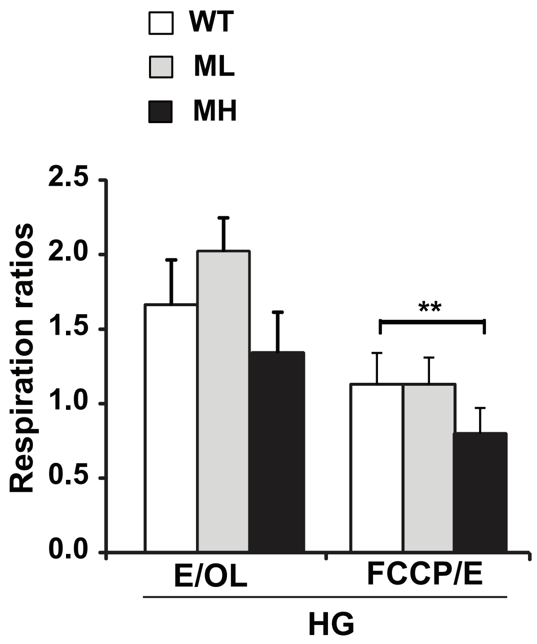

Figure S3. Measurement of the respiration ratios. To determine whether respiration is coupled to ATP production in ML and MH cells compared to WT cells, we measured the ratios of the endogenous to oligomycin-inhibited respiration (E/OL) and FCCP uncoupled to endogenous respiration (FCCP/E). No significant change in the E/OL and FCCP/E respiration ratios was observed in ML cells, however a significant decrease in the FCCP/E respiration ratio was observed in MH cells compared to WT cells indicating that the respiration is uncoupled from ATP production in MH cells.

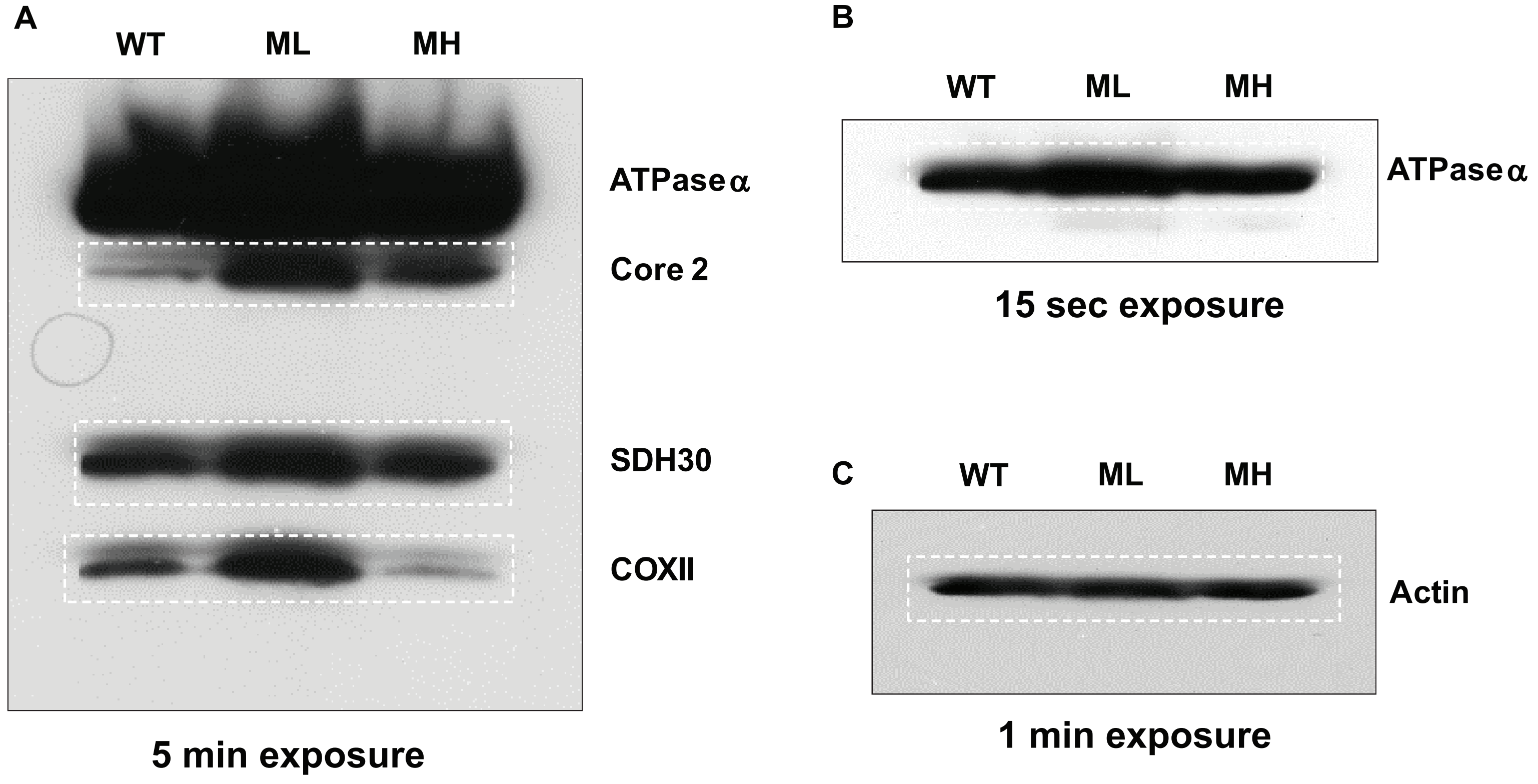

Figure S4. Western blot analysis of the OXPHOS proteins. (**A**) Total OXPHOS human monoclonal antibody cocktail (Abcam # ab110411) was used to detect the steady-state levels of ATPase α (~55 kDa), Core 2 subunit of complex III (~48 kDa), SDH 30 subunit of complex II (~30 kDa), and COXII subunit of complex IV (~26 kDa), respectively. Five minutes exposure of the blot is shown. (**B**) Shows a 15 seconds exposure of the blot to capture the ATPase α band signal. (**C**) A polyclonal antibody against actin (Sigma # A2066) was used as a loading control. The actin band (~42 kDa) is shown after a 1 minute exposure of the blot. Note: All cropped band images depicted by a white dotted rectangle are shown in Fig. 3C.

Supplementary Table 1. Human gene primer sequences used for qPCR analysis.

Gene	Primer sequence (5'- 3')
CYB_F	caatggcgcctcaatattct
CYB_R	gccgatgtttcaggtttctg
COXII_F	catccctacgcatcctttaca
COXII_R	gccgtagtcggtgtactcgt
ATP6_F	cctctacctgcacgacaaca
ATP6_R	ggtcattaggagggctgagag
18SrRNA_F	atccattggagggcaagtc
18SrRNA_R	gctcccaagatccaactacg
TFAM_F	gaacaactacccatatttaaagctca
TFAM_R	gaatcaggaagttccctcca
PGC1alpha_F	aatttttcaagtctaactatgcagacc
PGC1alpha_R	caaaatccagagagtcatacttgc
PGC1beta_F	cagacagaacgccaagcat
PGC1beta_R	tcgctggagagattttgaatg
PRC1_F	gcccctagaccggttacaag
PRC1_R	ggggcttccataactggtg
PPARalpha_F	gcactggaactggatgacag
PPARalpha_R	tttagaaggccaggacgatct
ERRalpha_F	ggcggcagaagtacaagc
ERRalpha_R	attcactggggctgctgt
COX15_F	ctgctggctttggcgtat
COX15_R	gagcctgactggtgagtgg
ATP5A1_F	tgctattggtcaaaagagatcca
ATP5A1_R	gtagccgacaccacaatgg
PC_F	ccctgctggtcaaagtcatt
PC_R	tgctggttgttgagcacatt
CPT1A_F	gacaatacctcggagcctca
CPT1A_R	aataggcctgacgacacctg

ACO2_F	gtcctggccaatgcttgt
ACO2_R	gttgtaggaggtgacgattgtg
B2M_F	agatgagtatgcctgccgtgtgaa
B2M_R	tgctgcttacatgtctcgatccca
bactin_F	ccaaccgcgagaagatga
bactin_R	ccagaggcgtacagggatag
